# Towards the Conservation of Endangered Avian Species: A Recombinant West Nile Virus Vaccine Results in Increased Humoral and Cellular Immune Responses in Japanese Quail (*Coturnix japonica*)

**DOI:** 10.1371/journal.pone.0067137

**Published:** 2013-06-25

**Authors:** Joanne A. Young, Wilfred Jefferies

**Affiliations:** 1 The Michael Smith Laboratories and the Department of Zoology, University of British Columbia, Vancouver, B.C., Canada; 2 Department of Microbiology and Immunology, Department of Medical Genetics, University of British Columbia, Vancouver, B.C., Canada; 3 Brain Research Centre, University of British Columbia, Vancouver, B.C., Canada; 4 Centre for Blood Research, University of British Columbia, Vancouver, B.C., Canada; Glasgow University, United Kingdom

## Abstract

West Nile Virus (WNV) arrived in North America in 1999 and is now endemic. Many families of birds, especially corvids, are highly susceptible to WNV and infection often results in fatality. Avian species susceptible to WNV infection also include endangered species, such as the Greater Sage-Grouse (*Centrocercus uropbasianuts*) and the Eastern Loggerhead Shrike (*Lanius ludovicianus migrans*). The virus has been shown to contribute towards the likelihood of their extinction. Although a clear and present threat, there exists no avian WNV vaccine available to combat this lethal menace. As a first step in establishing an avian model for testing candidate WNV vaccines, avian antibody based reagents were assessed for cross-reactivity with Japanese quail (*Coturnix japonica*) T cell markers CD4 and CD8; the most reactive were found to be the anti-duck CD8 antibody, clone Du-CD8-1, and the anti-chicken/turkey CD4 antibody, clone CT4. These reagents were then used to assess vaccine performance as well as to establish T cell populations in quail, with a novel population of CD4/CD8 double positive T cells being identified in Japanese quail. Concurrently, non-replicating recombinant adenoviruses, expressing either the WNV envelope or NS3 ‘genes’ were constructed and assessed for effectiveness as avian vaccines. Japanese Quail were selected for testing the vaccines, as they provide an avian model that parallels the population diversity of bird species in the wild. Both the level of WNV specific antibodies and the number of T cells in vaccinated birds were increased compared to unvaccinated controls. The results indicate the vaccines to be effective in increasing both humoral and cellular immune responses. These recombinant vaccines therefore may find utility as tools to protect and maintain domestic and wild avian populations. Their implementation may also arrest the progression towards extinction of endangered avian species and reduce the viral reservoir that potentiates infection in humans.

## Introduction

West Nile virus (WNV) is an insect borne pathogen passed in the blood meals of mosquitoes. It is currently endemic throughout North American and is of concern to avian, equine, reptile and human populations. WNV causes significant levels of morbidity and mortality in birds. Furthermore as birds are part of the amplification cycle for the virus they act as incubators serving to amplify the reservoir of the virus, this facilitates the spread of infection to humans and other species. This infection paradigm has risen as an emerging and expanding public healthcare risk. In bird species this has also been devastating for highly susceptible avian species, particularly those species that are also rare or endangered. Two such species are the Greater Sage-Grouse (*Centrocercus uropbasianuts*) and the Eastern Loggerhead Shrike (*Lanius ludovicianus migrans*). Greater Sage-Grouse are found in Alberta, Canada as well as several sites across the North Eastern U.S.A. When WNV arrived at many of these geographical locations it initially resulted in population declines. In fact, the survival rates in many of these infected sites fell by 25% to 56% compared to non-infected sites [1], resulting in what many termed, “The Silent Spring”. Later, following subsequent years of natural WNV infections, some Sage-Grouse were found to be positive for WNV antibodies, indicating that not all birds were dying. However the infection incidences, in the populations studied, were only 4% to 29%; this implies that most of the birds had not been exposed to WNV and were still susceptible to its pathogenic effects [2]. The Eastern Loggerhead Shrike is considered rare throughout North America, and to maintain population numbers two captive breeding colonies were set up. In 2002, five captive birds died shortly after contracting WNV and none of the remaining 37 birds tested positive for WNV antibodies, these data predict a 100% mortality rate for this species [3]. These cases highlight the requirement for an avian vaccine against WNV, which could be used to assist in preventing extinction of rare species of birds and could also be used to reduce the reservoir in intermediate species that facilitates infections in humans.

The WNV vaccines we have developed are based on a non-replicating recombinant adenovirus platform and were tested in Japanese Quail (*Coturnix japonica*) (referred to here as quail). This species was selected as a suitable animal model as they represent a relatively genetically diverse avian population, unlike many available lines of chickens. The use of quail would therefore more accurately reflect vaccine performance in the avian species that are the target populations for vaccination, namely wild or captive birds. As Japanese quail are not commonly used for detailed immunological studies, suitable reagents that would recognise immuno-markers within the quail, such as CD4 and CD8, had to be identified. Antibody reagents specific to other avian species were screened for cross-reactivity with quail cells. The two recombinant vaccines created contained either the WNV pre-membrane and envelope (pre-M/E) gene or NS3 gene. This was undertaken to investigate the possibility that a structural viral protein, such as the envelope protein, would elicit a different immune response compared to a non-structural viral protein, such as the NS3 protein.

## Methods

### Avian Antibody Screening

Randomly selected naive quail were euthanised by an inhalation overdose of isoflurane, and trunk blood was collected to 4 ml EDTA tubes, inverted 8 times and placed on ice. Spleens were harvested and placed in PBS on ice. Blood was diluted with an equal volume of PBS and layered over a volume of Ficoll-Paque Plus equal to that of the diluted blood. After centrifugation, cells were harvested from the ficoll/plasma interface. Cells were washed once in RPMI medium and resuspended in RPMI medium for counting. Spleens were mashed through a 70 µm mesh cell strainer and resuspended in RPMI medium for counting. Leukocytes from spleens and blood were identified and counted using a haemocytometer.

Cells were transferred to a 96–well round bottomed plate for antibody staining at 1×10^6^ cells per well. Antibodies were used at a concentration of 0.2 µg per 1×10^6^ cells, unless otherwise stated. Cells were spun and resuspended in antibody diluted in FACS buffer (PBS with 2% FBS). The cells were incubated with the antibody at 4°C for 30 minutes and washed twice in FACS buffer, prior to analysis by flow cytometry. Using Flowjo software, live cells were selected followed by the removal of autofluorescent cells from analysis; histograms of remaining cells were displayed on the relevant fluorescent channel for the conjugate used. All antibodies screened were purchased from Abcam.

### Assessment of T Cell Populations in Quail

Four randomly selected naive quail were used for each repeat of the experiment, with cells being harvested and prepared for antibody staining as for the antibody screen. For CD8, the duck CD8α specific Du-CD8-1 mAb (Abcam, #ab41327) was used and for CD4, the anti-chicken/turkey CT-4 mAb (Abcam, #ab25345) was used. Antibodies were diluted in FACS buffer; Du-CD8-1 was used at 0.1 µg per 1×10^6^ cells and CT4 at 0.2 µg per 1×10^6^ cells. For double staining of cells, the same amounts of both antibodies, as used for single staining, were added to the same well. Unstained control wells received FACS buffer only and the plate was left at 4°C for 30 mins. Cells were then washed and analysed by flow cytometry as for antibody screening.

### Vaccine Construction

The recombinant adenovirus vaccines were created using the Admax™ Adenoviral Vector Creation System from Mircobix Biosystems Inc. The system included an adenovirus genomic plasmid and a shuttle plasmid called pDC316. The WNV DNA was inserted into the shuttle and the recombinant viruses created through recombination of the two plasmids in ‘293’ cells (293[HEK-293], #CRL-1573, from ATCC, referred to here as ‘293’ cells).

WNV DNA, for insertion into pDC316, was created from inactivated WNV RNA from an infected bird, a gift from Mike Drebot of the Public Health Agency of Canada in Winnipeg. The RNA was extracted using the Qiagen RNeasy Mini Kit, RNA was converted into cDNA by use of the Invitrogen Superscript II RT kit, 20 ng of RNA, was used in the standard protocol which included 2 pmole of gene specific primers (3′ end primers only) for the WNV ‘genes’. Individual reactions were set up that included either primers for NS3 or for the pre-membrane ‘gene’ from WNV. The cDNA was stored at −20°C until required.

Specific PCR primers were designed for synthesis of the premebrane-env (preM-E) or NS3 DNA to be inserted into pDC316, (preM-E fwd: GCATCTTAAGTACCAATGGGAGAGATT, preM-E rev: GCATTCTAGATTAAGCGTGCACGTTCA, NS3 fwd: AGGAACTTAAGTACCCTCCGCACAACAC and NS3 rev: AGAAAGATCTTTAACGTTTTCCCGAGG). PCR reactions were set up using Invitrogen Platinum Pfx DNA Polymerase, per 0.2 ml PCR reaction tube: 5 µl 10× Pfx buffer, 5 µl 10× PCRx enhancer, 1.5 µl 10 mM dNTP mix, 1 µl 50 mM MgSO_4_, 1 µl 0.1 mM primer for both forward and reverse priming, approx 100 ng of the relevant cDNA template and 0.5 µl Pfx polymerase, with the volume being totalled to 50 µl with nanopure water. Cycler conditions were: 1 cycle of 94°C for 5 min, 35 cycles of 94°C for 15 sec and 68°C for 2 min and 15 sec, 1 cycle of 68°C for 10 min. PCR products were run on a 1% agarose gel and bands of the correct sizes were gel purified using the QiaexII Gel Extraction kit (Qiagen).

Restriction enzyme digests of DNA for both of the WNV inserts and pDC316 were performed using both *Bgl*II and *Eco*RI, reactions were then purified using the QIAquick PCR purification kit. Ligation reactions were set up, using T_4_ ligase (Invitrogen), to insert the WNV DNA into pDC316. After incubation, ligation reactions were used to transform *E.coli* for DNA amplification. Bacterial colonies were selected and screened for the presence of the WNV DNA using PCR, with the same primers and conditions as for creating the WNV insert DNA. Positive colonies were re-selected from the plates and used to amplify further DNA.

Co-transfection of ‘293’ cells were undertaken, using Lipofectamine and Plus reagent (Invitrogen), including DNA from the adenovirus genomic plasmid and pDC316 containing either the pre-M/E or NS3 WNV DNA, to create the recombinant adenovirus vaccines, rAdE or rAdNS3 respectively. The genomic plasmid was also co-transfected with pDC316 only, as an empty shuttle plasmid, to create the empty vector control recombinant adenovirus, rAdMT. Briefly, co-transfections were set up using ‘293’ cells in 6 well plates at 50% confluency, DNA and reagents for each well of the plate were mixed individually in microtubes prior to addition to the cells. Per microtube: 6 µl plus reagent were added to 2 µg of each relevant DNA diluted in a total of 100 µl transfection medium (serum free DMEM), 15 min later 4 µl of lipofectamine diluted in 100 µl transfection media were added to each tube, followed by a second 15 mins incubation. Cells were washed once with transfection medium, prior to addition of the DNA containing transfection medium. Following a 3 hour incubation the transfection medium was removed and the cells were overlaid with an agarose overlay (equal volumes of 1% seaplaque agarose in nanopure water and 2X DMEM with 4% FBS), plates were returned to the incubator. 2 ml additional overlay were added to each well at 6 days post transfection. At 14 day post transfection plaques were visible and large enough to harvest into 1.5 ml DMEM (2% FBS), to make a crude virus stock, for use to further amplify the adenovirus vaccines. Three rounds of plaque purification were undertaken to ensure clonality of each vaccine prior to large scale amplification of the vaccines to create working vaccine stocks.

Adenoviral DNA was isolated for analysis following the protocol in Current Protocols in Human Genetics, unit 12.4, where the viral capsid is digested with proteinase K prior to isolation and purification of the DNA. Adenovirus DNA was screened, using PCR and the same primers as used to create the inserts, to ensure that either pre-M/E or NS3 WNV DNA was present.

Adenovirus stocks, for use in vaccine testing, were then amplified in ‘293’ cells, crudely extracted, and then purified using a caesium chloride gradient. The caesium was then removed from the adenovirus by dialysis in adenovirus buffer A195 [4], using slide-a-lyser cassettes (ThermoScientific) and placed at −80°C for storage until required. The Adeno-X Rapid Titre Kit (Clontech) was used to quantify the infectivity of each batch of vaccine produced, resulting in infectivity being measured in IFU/ml (infection forming units per ml).

### West Nile Virus Antigen

WNV antigen from WNV infected suckling mouse brain was a gift from Ms. A. Dilbernardo of the National Microbiology Lab, Winnipeg. Lyophilised samples were reconstituted in nanopure water and protein content was quantified using a BCA assay. The antigen was used directly in the serum ELISA. To prepare the antigen for use in the IFN-γ assay, antigen was diluted to 1.4 mg/ml in RPMI, and sonicated on ice at 30 W for 15 sec, left for a 2 min rest and then received another sonic burst at 60 W for 30 sec.

### Vaccine Assessment

**Figure 1:**
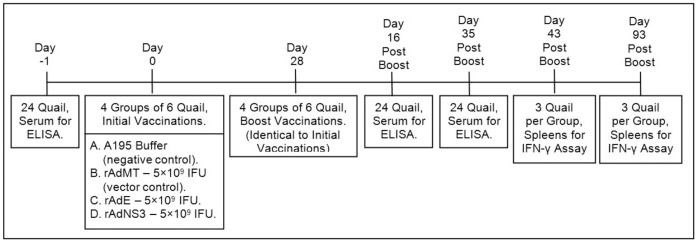
Timeline with Details of Vaccinations and Samples Taken. The entire trial was run over a period of 122 days. Blood samples were first taken the day before any birds were vaccinated; this was followed by initial vaccinations and then booster vaccinations 28 days later. Blood and spleen samples were then collected over the 93 day period following the boost injections.

Quail were divided into four groups of six birds each, with each group receiving one of four different vaccinations. A negative control group received A195 buffer alone, an empty vector control group received rAdMT and the two vaccination groups received either rAdE or rAdNS3. Birds were vaccinated intramuscularly, into the breast muscle, with 5×10^9^ IFU of the relevant vaccine, in a total volume of 200 µl using additional A195 buffer, or with 200 µl of buffer alone for the negative control group. All birds received a second, identical injection 28 days post vaccination. Blood, for serum, was collected from all birds one day prior to initial vaccination (day −1) and again at days 16 and 35 post boost for all birds. Two birds from each group were euthanised on days 43 and 93 post boost, and spleens collected for analysis by IFN-γ assay. See Figure 1 for a timeline.

### Vaccine Assessment - IFN-γ Assay

Birds were euthanised following an isoflurane overdose; spleens were harvested and placed immediately in PBS on ice. Spleens were disaggregated into single cell suspensions by pressing them through 70 µm nylon cell strainers, into RPMI medium. Cells were placed on ice and counted, using a haemocytometer, to obtain a count of cells with a lymphocyte morphology. To set up the IFN-γ assay for lymphocyte re-stimulation, splenocytes from each sample were placed into round bottomed 96-well tissue culture plates, re-stimulation treatments were set up in duplicate. For each sample, 1×10^6^ cells were added to relevant wells of each plate, and cells were washed once. Cells were re-suspended in fresh RPMI medium, with or without other components, to set up the following re-stimulation conditions: (i) No re-stimulation (negative control) –200 µl RPMI medium alone were added to each of the relevant wells; (ii) E/3 re-stimulation –rAdE and rAdNS3 vaccines were diluted in RPMI medium to 1×10^5^ IFU virus in 200 µl. For samples from birds that received either the negative control injection or the rAdMT vaccine, 100 µl of diluted rAdE (5×10^4^ IFU) and 100 µl of diluted rAdNS3 (5×10^4^ IFU) were added to each of the relevant wells. For samples from birds that received either rAdE or rAdNS3 vaccines, 200 µl (1×10^5^ IFU) of only the same vaccine as the bird was injected with, were added to each of the relevant wells; (iii) WNV re-stimulation –WNV antigen was diluted to 140 µg/ml in RPMI medium and then 100 µl were added to each of the relevant wells. Note, WNV re-stimulation was not applied to any samples taken on day 43 post-boost.

Two plates were set up with identical conditions, one to be used to identify CD8 cells and one for CD4 cells, plates were incubated at 39°C, 5% CO_2_ for 2 days. Golgistop™ (BD Bioscience), diluted in RPMI medium, was added to the media already on the cells, to a final concentration of 1.5 µl/ml. Plates were gently shaken laterally, to mix and returned to the incubator for 6 hrs.

For antibody staining the Cytofix/Cytoperm™ Plus Kit (BD Bioscience) was used to fix and permeabilise the cells, in conjunction with specific antibodies. For CD8, the duck CD8α specific Du-CD8-1 mAb (Abcam, #ab41327) was used; for CD4, the anti-chicken/turkey CT-4 mAb (Abcam, #ab25345) and for IFN-γ, the anti-chicken IFN-γ pAb (Genway, #18-738-78426) was used. Prior to use CD4 and CD8 antibodies were conjugated to APC and the IFN-γ antibody to Alexa-488 by the AbLab at UBC. All antibodies were used at the concentration of 0.2 µg per 1×10^6^ cells. Plates were removed from the incubator and washed once with 200 µl FACS buffer (2% FBS in PBS) per well. For cell surface staining of CD markers, CD4 or CD8 antibodies were diluted in 100 µl FB per well, plates were left on ice for 30 min and then washed once as before. Cells were then fixed and permeabilised using the Cytofix/Cytoperm™ kit and standard protocol. To stain any intracellular IFN-γ, the IFN-γ antibody was diluted in 1× perm/wash buffer and added to all wells, except relevant control wells, in a volume of 100 µl per well, plates were then left on ice for 30 mins under foil. Plates were washed twice with 1× perm/wash buffer and once with FACS buffer prior to processing by flow cytometry. Subsequently Flowjo software was used to select live cells, remove cells with high autofluorescence and, in conjunction with single stained control samples, to quantify the percentage of remaining cells that were either CD8+, CD8+ and IFN-γ+, CD4+ or CD4+ and IFN-γ+.

### Vaccine Assessment – Serum ELISA

Approximately 200 µl of blood were collected from the brachial wing vein of each bird, into capillary tubes, tubes were held at room temperature to allow clotting and spun to isolate serum. 3 µg of WNV antigen in 0.05 M carbonate buffer (pH 9.6) were added to relevant wells of a flat bottom 96-well ELISA plate. Plates were washed (0.1% tween-20 in PBS) 5 times, blocked (5% milk powder in PBS) at 37°C for 40 min, followed by another 5 washes. Two-fold serial dilutions of serum, from 1∶20 to 1∶160, in blocking buffer were set up, a positive control well was included using serum from a WNV infected goose at 1∶200 dilution and 2 negative control/background wells with no serum were also included. Plates were incubated at 37°C for 2 hr and washed as before. An anti-bird IgG HRP conjugated antibody (Benthyl Labs, concentration 1 mg/ml) diluted 1∶600 in blocking buffer was added and plates incubated for 1 hr then washed 5 times. SureBlue Reserve Peroxidase Substrate (KPL) was warmed to room temperature prior to use, this was added to all wells and left until the colour was deemed suitably dark, usually 5 mins. The reaction was stopped by adding an equal volume of 1N sulphuric acid and the plate was then read at 450 nm on a plate reader, within 15 min.

### Statistical Analysis

#### IFN-γ assay

Results were initially assessed using Flowjo software, the number of positive cells were then analysed using a specially written script in the R program (see Protocol S1). The script used a generalised linear mixed model, using Poisson regression and taking into account both fixed and random effects to assess the data. For individual birds from each vaccination group, the percentage of cells that received re-stimulation treatment was compared to those that received no re-stimulation treatment. These comparisons were run separately for each CD marker, with and without IFN-γ positive cells, within each vaccination group, including the negative control group.

#### Serum elisa

A paired-T test analysis was undertaken using Minitab v16 Statistical Software, pairing OD from either day 16 or day 35 with OD readings from day −1, for each individual bird and for all serum dilutions. Mean OD values for all serum dilutions within each day and vaccination group combination were also calculated.

### Animal Ethics

All birds were housed and handled, and experiments run, in accordance with guidelines laid down by the Animal Care Committee of the University of British Columbia. The committee issued certificate number A08-0628 as approval of all work undertaken.

## Results

Vaccine studies in captive bird populations are rare and problematic. As Japanese quail are not commonly used for detailed immunological studies, suitable reagents that would recognise immune markers within the quail, such as CD4 and CD8, had to be identified.

### Avian Antibody Screening

Four anti-CD8 and three anti-CD4 avian antibodies were screened for cross-reactivity with quail. For CD8, the antibodies, CT8, 3-298 and EP72 (chicken CD8α reactive) and Du-CD8-1 (duck CD8α reactive) were assessed, and for CD4, the antibodies EP96 (chicken reactive), CT4 (chicken/turkey reactive) and Du-CD4-1 (duck reactive). Of these, the CD4 antibody EP96 and the CD8 antibodies CT8, EP72 and 3-298 did not demonstrate recognition of quail CD4 or CD8 respectively. Initial testing indicated that the CD4 antibody Du-CD4-1 did recognise quail CD4 (data not shown), however a subsequently tested antibody, CT-4, provided more defined fluorescence profiles following staining, so no further testing of Du-CD4-1 was undertaken.

**Figure 2:**
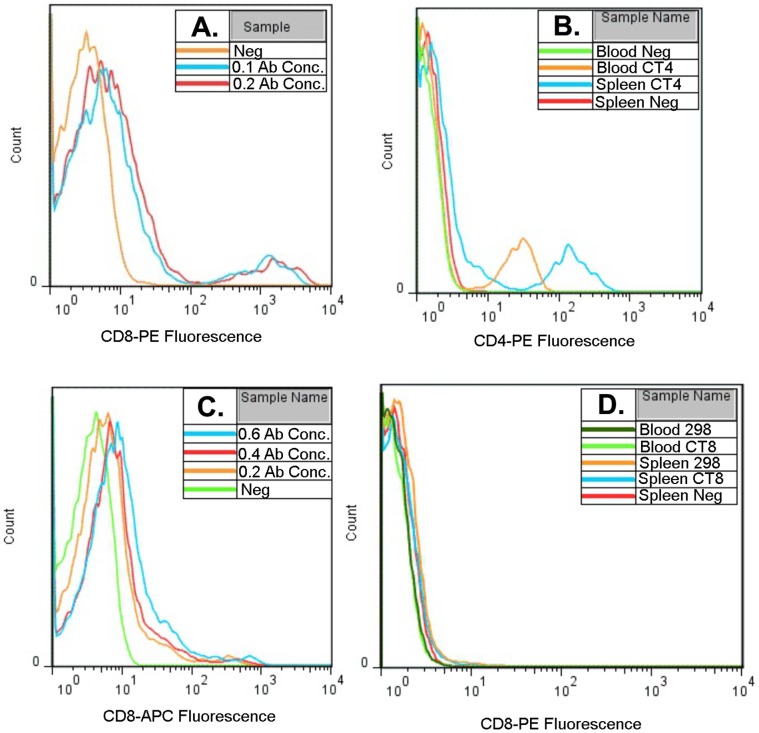
Avian Antibody Cross-Reactivity Screen for Quail CD4 and CD8. Examples of histograms of fluorescence for cells from individual quail stained with different avian antibodies. Cells were previously gated to select live cells and remove autofluorescent cells. **A.** Shows quail splenocytes stained with 2 different concentrations of the CD8 antibody Du-CD8-1, 0.1 µg/10^6^ cells (0.1 Ab conc.) and 0.2 µg/10^6^ cells (0.2 Ab conc.) and an unstained control (Neg). The antibody does bind quail CD8 and although the lower concentration shows slightly lower levels of fluorescence the number of positively stained cells is very similar for both concentrations. **B.** Shows leukocytes from blood and spleen of quail stained with the CD4 antibody CT4 (CT4 samples) or unstained (Neg). The antibody binds to quail CD4, although the level of fluorescence is lower in the blood than the spleen, the number of positively stained cells is similar for both sample types. **C.** Shows quail splenocytes stained with three different concentration of the CD8 antibody EP72, 0.6 µg, 0.4 µg and 0.2 µg/10^6^ cells as samples 0.6, 0.4 and 0.2 Ab conc. respectively. Although staining with the antibody appears to increase fluorescence levels for the negative population, when compared to the unstained sample (Neg), there was no clearly defined positive population of cells. It was considered that this antibody did not recognise quail CD8. **D.** Shows leukocytes from blood and spleen of quail stained with either CD8 antibody 3–298 or CT8, as samples 298 or CT8 respectively, as well as an unstained spleen sample (Spleen Neg). As fluorescent levels are all similar to the unstained sample it was concluded that neither of these antibodies recognised quail CD8.

Both the CD8 antibody Du-CD8-1 and the CD4 antibody CT4 demonstrated binding to quail CD8 and CD4 respectively, as seen in the fluorescent antibody binding profiles. Figure 2 shows fluorescence profiles from both cross-reactive antibodies and examples from the other antibodies tested.

### Assessment of T Cell Populations in Quail

CD8 and CD4 antibody staining of leukocytes from both blood and spleens of quail were repeated four times, using Du-CD8-1 and CT4 antibodies respectively, although blood leukocytes could not be obtained for all experiments. Range and means of the percentage of cells positive for either CD8 or CD4 are shown in Table 1, as well as overall means for each cell population. For quail, overall percentages of CD8+ cells constitute 12.5% of blood cells and 31% of splenocytes and CD4+ cells 0.9% of blood cells and 3.7% of splenocytes. As further assessment of the quail spleen samples, standard deviation and the coefficient of variation were also calculated. For CD8+ cells these values were 5.093 and 16.5% respectively and for CD4+ cells 2.313 and 62.9% respectively. There were insufficient data to make the same additional calculations for the blood samples.

**Table 1:** Percentages of CD4 and CD8 Positive Cells in Blood and Spleens of Quail.

	CD4+ PBL	CD4+ Spleen	CD8+ PBL	CD8+ Spleen
#1.	N/D	6.9	N/D	33.7
# 2.	0.9	3.3	7.1	29
# 3.	N/D	3.1	N/D	24.8
# 4.	0.8	1.4	17.8	36.3
**Overall Mean**	**0.85**	**3.7**	**12.45**	**31.0**
**Range of Percentages**	**0.147–1.83**	**0.237–8.39**	**2.9–26.4**	**15.0–46.2**

The mean numbers of CD4 or CD8 positive cells are shown for four experiments (#1–4) using quail splenocytes (spleen) and two experiments (#2 and #4) using peripheral blood leukocytes (PBL). Overall mean is the mean for all experiments, for each cell type and cell marker. The range of percentages shows the range of percent of positive cells seen for individual birds from all experiments. N/D – not done.

**Figure 3:**
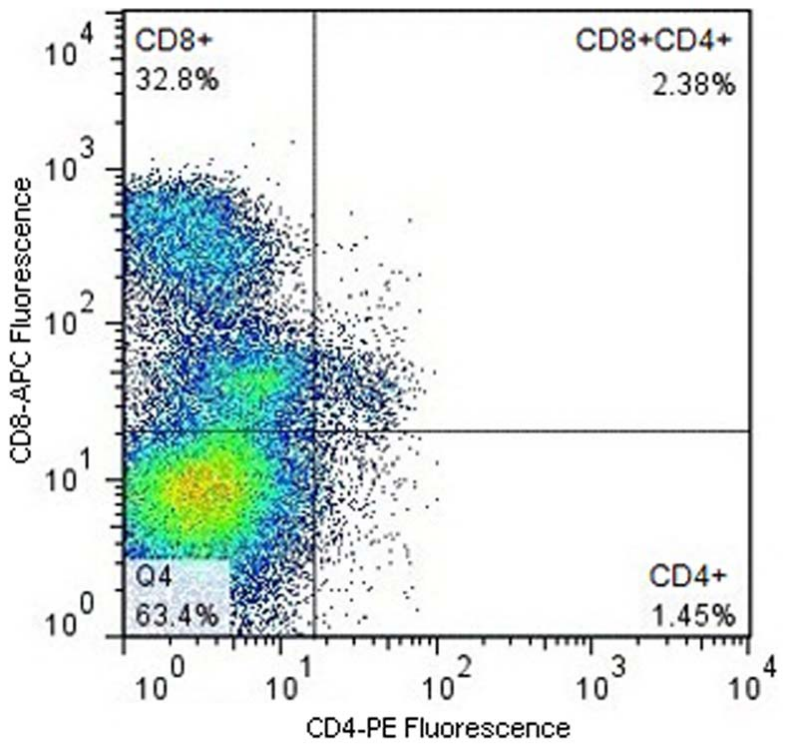
Fluorescent Profile of Quail Splenocytes including CD4/CD8 Double Positive Cells. Quail splenocytes were stained concurrently with both Du-CD8-1 (CD8) and CT4 (CD4) antibodies and were gated to select live cells and remove autofluorescent cells. The example shown, from a single bird, consists of 32.8% CD8+ cells, 1.45% CD4+ cells and 2.38% CD8+/CD4+ cells. The CD8+ population is subdivided into two populations with either a high or low level of CD8 expression, only the CD8+low population contains cells that are also CD4+.

Splenocytes were also stained concurrently with CD8 and CD4 antibodies and a population of cells that were double positive for both T cell markers was identified. Mean averages for each population were calculated: 39% were CD8+ only, 1.71% were CD4+ only and 2.6% were CD4+CD8+ double positive cells, giving an overall CD8+ of 41.6% and CD4+ of 4.31%. The fluorescent profile of cells from one of the quail can be seen in Figure 3.

### Vaccine Assessment - IFN-γ Assay

Graphical output from the R script (see Figure S1) shows numbers of T cells and numbers of IFN-γ+ T cells for all the different vaccine/re-stimulation combinations from both days tested. Table 2 shows all significant differences between percentages of positively stained T cells for either re-stimulation treatment compared to no re-stimulation treatment for each vaccination group. There were no significant differences within the A195 buffer only group (negative control). For the empty vector (rAdMT) vaccinated group, there were slightly more CD4+ cells following both re-stimulation treatments. For the rAdE vaccinated group, there were more CD4+ cells and CD4+IFN-γ+ cells with WNV antigen re-stimulation. The rAdNS3 vaccinated group showed more CD4+ and CD8+ cells when re-stimulated with E3 vaccine, and more CD4+IFN-γ+ cells, with WNV antigen re-stimulation.

**Table 2:** Significant Differences Between Re-Stimulation Treatments within Vaccination Groups.

Vaccination Dose (5×10^9^ IFU)	A195		rAdMT		rAdE		rAdNS3	
Re-Stimulation	E3	WN	E3	WN	E3	WN	E3	WN
All CD4+	–	–	*	*	o	**	*	*
All CD8+	–	–	–	–	–	–	–	–
CD4+ IFN-γ+	–	–	–	–	–	***	-	*
CD8+ IFN-γ+	–	–	–	–	–	–	–	–

For each vaccination group, significant differences between either of the re-stimulation treatments and the no treatment are shown with P values indicated. ‘-’ = no difference, ‘o’ = P<0.1, ‘*’ = P<0.05, ‘**’ = P<0.01, ‘***’ = P<0.001. A195 is the buffer only control, rAdMT is the empty vector control and rAdE, rAdNS3 are the two variations of the vaccine. Both vaccinated groups show increases in more cell types than for either control group, for both re-stimulation treatments, although no increase in CD8+IFN-γ+ cells was seen in either group. Some increase in CD4+ cells was seen in the empty vector control group, but none in the buffer only control group.

### Vaccine Assessment - Serum ELISA

Antibody levels were assessed using a WNV specific ELISA, with a paired-T test analysis run to compare OD values between days. All results from the paired-T test were significant, indicating an overall increase in antibody levels, over time, for all groups. The difference between the mean OD for day-1 and the mean OD for either day 16 or 35, for each vaccination group were calculated (Table 3). The rAdE-vaccinated group demonstrates the highest levels of WNV antibodies on both days tested, as indicated by the largest difference in means. By day 35 the rAdNS3 vaccinated group had the second highest difference in means, however they are not significantly higher than either control group. Also, for both rAdE and rAdNS3 differences are less on day 35 than on day 16, indicating that levels of antibody, in both groups, had fallen between days 16 and 35.

**Table 3:** Significant Differences of WNV Antibody Levels.

Day	Vaccination Group	Means of Diffs.	SEM	Rank of Mean Diffs.
16	rAdE	0.14	0.041	1
16	rAdMT	0.054	0.011	2
16	rAdNS3	0.049	0.013	3
16	A195	0.037	0.012	4
35	rAdE	0.058	0.016	1
35	rAdNS3	0.019	0.0040	2
35	A195	0.018	0.0035	3
35	rAdMT	0.017	0.0037	4

Means of Diffs. is the mean of the difference of all paired OD values, between reading taken prior to vaccination (day −1) and those taken on either day 16 or day 35 (as specified in Day column), for each serum dilution, for all birds within each vaccination group. A195 is the buffer only control, rAdMT is the empty vector control and rAdE, rAdNS3 are the two variations of the vaccine. SEM – standard error of means. Rank Of Mean Diffs. places the difference of means in order, for each day, with the largest value as 1. For days 16 and 35 the rAdE vaccinated group produced the highest level of WNV antibodies, as indicated by the significantly larger difference compared to the second highest group for each day.

## Discussion/Conclusions

### Avian Antibody Specificity

None of the antibodies used to assess the immune responses in this study were species specific for quail. Cross-reactivity of the antibodies to quail cells was shown by the presence of positively stained cells, indicating that the antibodies were binding to the cells. During this study specificity of binding was not directly tested but we believe T cell marker and IgG/Y binding was likely to be specific in quail and this is reflected in our conclusions. Ideally quail specific antibodies should be used for complete accuracy or further studies undertaken to confirm antibody specificity. However non-quail antibodies would be valid for use in quail for comparative purposes, such as their use in the current vaccine assessment.

Neither IgG/Y, CD4 nor CD8 have been sequenced for quail and so were unavailable for comparison to sequences relating to the antibodies used. However, quail IFN-γ has been sequenced (NCBI Accession #AJ001678) and when compared to the chicken IFN-γ sequence (NCBI Accession # DQ470471) showed 95% similarities. Therefore it is likely that the anti-chicken IFN-γ antibody used was also recognising and binding quail IFN-γ.

### Avian Antibody Screening

Of the seven avian antibodies screened for cross-reactivity with quail T cells (four for CD8 and three for CD4), one cross-reacted with CD8 (Du-CD8-1) and two with CD4 (Du-CD4-1 and CT4), although one of these (Du-CD4-1) was only an initial assessment. This cross-reactivity may indicate conserved, or partially conserved, regions in the CD molecules, between the species the antibodies were specific for, namely duck or chicken, and quail. It is possible that the antibodies are binding to different epitopes of the CD molecules in quail than in chicken or duck. However, that the antibodies do bind to quail T cell markers demonstrates their usefulness as a tool for making more detailed immunological studies possible in quail.

### Assessment of T Cell Populations in Quail

T cell populations, previously established in chickens are shown below, for comparison to the quail values obtained from this study, which are shown in brackets for each cell population. T cell populations in chicken spleens [5] were found to be 53–55% (31%) CD8+ cells and 5–7% (3.7%) CD4+ cells. While chicken PBMCs [6] consisted of 11–19% (12.5%) CD8+ cells and 1.6–3.4% (0.9%) CD4+ cells. Overall values for quail are very similar, although slightly lower, than for chickens.

To further validate the quail T cell spleen data, calculations were made to quantify the variation between experiments using the same antibody. The calculated means of CD positive cells showed a higher range and larger standard deviation for the CD8 antibody compared to the CD4. However, as the mean for CD4+ cells was much lower than for CD8+ cells, a more comparable measure, as it is expressed as a ratio of each mean, is the coefficient of variation. This was much lower for the CD8+ cells (16.5%) than for the CD4+ cells (62.9%), indicating that values for the CD8 antibody may be more accurate than for the CD4 antibody.

During these experiments a small population of spleen cells that bound to both the CD4 and the CD8 antibodies was discovered. The phenotype of these cells was CD4+CD8+^l^°^w^ and to the best of our knowledge this is the first time that such a population has been discovered in Japanese quail. Populations of CD4/CD8 double positive T cells have previously been reported in chickens, as well as pigs, monkeys and humans [7]. In chickens the double positive cells were found to be functionally active CD4 cells that had reacquired the ability to expressed CD8α, but not CD8β, after leaving the thymus [8]. This CD8 expression pattern gave the chicken double positive cells the same phenotype, namely CD4+CD8+^l^°^w^, as seen in the quail. It is likely that the double positive cells in quail have the same function and origin as those in chickens. However, due to the preliminary nature of the data and the use of non-quail specific antibodies in the current study, further assessment of this population of cells in quail would be necessary to confirm our findings.

As cross-reactive antibodies were used, they may be binding to different epitopes in quail than in the species for which they are directly reactive, namely chicken or duck, and thus they may also bind with a different affinity. If the affinity was reduced in quail it could result in sub-optimal quantification of T cell numbers, either in initial binding or through subsequent handling of samples following staining, such as washes to remove excess antibody. This may explain the increased variation seen in the CD4 stained samples and may also contribute to the slightly lower numbers of T cells seen in quail when compared to chicken.

### Vaccine Assessment - IFN-γ Assay

The number of T cells present in each sample was calculated, as an indicator of T cell expansion and immune response initiation. However, in itself T cell number is not sufficiently indicative of an increased response, so the number of T cells producing IFN-γ was also calculated as an indication of T cell activation. Both numbers were calculated following *ex vivo* re-stimulation of the cells, to assess to what extent the vaccines were activating T cells specific for WNV or adenovirus antigens. Some T cells did produce IFN-γ in response to re-stimulation with rAdMT, indicating that the adenovirus itself was triggering a low level of immune response in the birds. However, the higher level of response seen in the rAdE vaccinated birds and the increased range of cells triggered in the rAdNS3 vaccinated group, indicates that the vaccines were also inducing a WNV specific IFN-γ response in the birds.

The vaccines trigger a better CD4+ cell response than CD8+ response, with only one CD8+ population, from the rAdNS3 vaccination/E3 re-stimulation group, significantly increasing, compared to 6 positive responses in CD4+ cells for both vaccinated groups. The rAdE vaccination group shows a very strong response in activating CD4+ cells to produce IFN-γ when re-stimulated with the WNV antigen.

### Vaccine Assessment - Serum ELISA

Birds for each vaccination group were housed in separate pens but within the same room, which may have allowed for a low level of cross-contamination of WNV antigens between groups. This would explain the increase in WNV specific antibodies seen in all groups during the 35 days following vaccination boost. However, the rAdE vaccinated group showed the highest increase in antibody levels, indicating that the vaccine was directly responsible for inducing antibody production. The rAdNS3 vaccine did not appear to trigger such a robust antibody response, as antibody levels were similar to the background levels of increase seen in the control groups.

The fact that the rAdE vaccine, which produces an antigen for a WNV structural protein, produces a more robust antibody response compared to the internal virus protein of the rAdNS3 vaccine, may reflect how quail cells process the WNV DNA region of the vaccines. The preM-E DNA, of the rAdE vaccine, contains DNA that codes for the transmembrane region of the envelope protein. It is likely that the pre-M-E protein is co-translationally transcribed into the endoplasmic reticulum (ER) membrane of the cell, as occurs in a WNV infected cell [9]. So in a rAdE vaccinated bird, the entire envelope protein could be expressed on the surface of a cell, thus allowing B cells to bind to any available epitope on the molecule. In contrast, the NS3 protein would likely be made directly in the cytosol of a cell expressing the rAdNS3 vaccine, based on studies of WNV infected cells [9]. Thus NS3 from the vaccine would be more likely to be processed into antigen for presentation via the classical MHC I pathway, and would therefore be more likely to trigger a CD8+ T cell response.

### Overall Vaccine Assessment

Overall the rAdE vaccine appears to perform better than the rAdNS3 vaccine, especially in inducing an antibody response, which may be due to the nature of the antigen being processed from the vaccines. As previously mentioned, vaccination with rAdE likely resulted in the complete envelope protein being available as antigen for B cells; this would lead to activation of any WNV specific B cell that could bind to any part of the protein. Vaccination with rAdE also triggered activation of more WNV specific CD4+ T cells, which would be required to fully activate the WNV primed B cells to produce antibodies. These cellular and humoral responses combined to generate a more robust antibody response to the rAdE vaccine than to the rAdNS3 vaccine.

The vaccines were tested in an avian species to more accurately mimic a genetically diverse avian population, this was to reflect the final target population for the vaccines, namely that of endangered birds. Quail are known to demonstrate more genetic diversity, 75–82% similarity within the same breeding lines [10], than the common avian immune model, the chicken, with 81–96% genetic similarity within breeding lines [11]; this was one reason quail were selected for vaccine assessment. However, although such aims were consistent with the wild immunology concept of reflecting a more relevant ‘real world’ situation for vaccine testing [12], in this instance it also meant a larger variation in the immune responses to the vaccines between individual birds. This contributed to a reduction in the clarity of results that may otherwise have been expected if a more standard animal model of immunity had been used. However, we were still able to show that the vaccines induced a significant CD4+ T cell response, as well as the rAdE vaccine producing a better antibody specific response.

### Final Comments

WNV is a serious threat to the survival of susceptible avian species in North America. An effective vaccine is needed in order to ensure long term viability. Furthermore, susceptible bird populations act as an intermediate reservoir for the infection of humans and other species and this paradigm has emerged as significant public healthcare risk. The vaccine candidates we describe may be effective in reducing the reservoir in birds and thereby reducing the threats to humans and other species but require validation in clinical trials assessing their ability to protect birds from lethal WNV challenges.

## Supporting Information

Figure S1 Graphical Output of Raw Data Obtained From R Script. The plots produced by the R script are shown below. T cell numbers were calculated for both total T cells (All) and IFN-γ positive cells (IFN-g+) for each day (days 43 and 93 post-boost (PB)) and each vaccine group/re-stimulation treatment combination tested. The combinations of vaccination and re-stimulation treatment are shown on the x axis. For ‘vaccine’, Neg is the negative control group, MT is rAdMT vector control group and Env and NS3 are for the groups vaccinated with rAdE and rAdNS3 respectively. The ‘Re-Stim’ indicates the re-stimulation treatment applied to the cells *ex-vivo*, and NS, E3 and WN denote the negative control (no treatment), E/3 and WNV antigen re-stimulation treatments respectively. The asterisks, just above the x axis, denotes any significant difference between that vaccine/re-stimulation combination and the corresponding vaccine/no re-stimulation treatment. *** indicates a P value of <0.001, ** a P value of <0.01, * a P value of <0.05 and ‘o’ a P value of<0.1. For all plots, the 2 points on each vertical line indicate T cell numbers from the 2 individual birds, used at that time point, from each of the 4 vaccination groups; where there appears to be only 1 point on a vertical line the values for each bird are very similar. On plots for day 43 there are 2 points for each bird to reflect the 2 re-stimulation treatments used and on plots for day 93 there are 3 points for each bird to reflect the 3 re-stimulation treatments used.
(DOC)

Protocol S1 R Script Used To Assess T Cell Numbers. The R script specially written to assess the numbers of T cells from all samples is shown below. For all cell types assessed, it compared cell numbers in re-stimulated samples to those in the no re-stimulation sample for individual birds in each experiment. The script used a generalised linear mixed model, using Poisson regression and taking into account both fixed and random effects on the data. Output from the script included calculations of P values for each comparison and plots of T cell numbers. Within the script certain lines were edited as appropriate: axis limits for the plots were set to suitable values to allow all data points, and the differences between them, to be seen clearly and for easier comparisons between cells types; whether 3 or 4 re-stimulation treatments had been used; whether there were 3 or 4 vaccine groups being analysed and whether the plots would show IFN-γ positive T cells only (denoted TR for T cell responding) or all T cells (TR cells plus IFN-γ negative cells, denoted TNR for T cell not responding).
(DOC)
