# Correction: Towards the Conservation of Endangered Avian Species: A Recombinant West Nile Virus Vaccine Results in Increased Humoral and Cellular Immune Responses in Japanese Quail (*Coturnix japonica*)

**DOI:** 10.1371/journal.pone.0099472

**Published:** 2014-06-03

**Authors:** The PLOS ONE Staff

The co-author, Dr. Joanne A. Young’s name has been changed to Dr. Jay A. Young.
