# Supplementary material for: Towards the Conservation of Endangered Avian Species: A Recombinant West Nile Virus Vaccine Results in Increased Humoral and Cellular Immune Responses in Japanese Quail (Coturnix japonica)
Source: PLoS One. 2013 Jun 25;8(6):e67137. doi: 10.1371/journal.pone.0067137 (PMC3692427; doi:10.1371/journal.pone.0067137)
Supplement: Figure S1 — Graphical Output of Raw Data Obtained From R Script. The plots produced by the R script are shown below. T cell numbers were calculated for both total T cells (All) and IFN-γ positive cells (IFN-g+) for each day (days 43 and 93 post-boost (PB)) and each vaccine group/re-stimulation treatment combination tested. The combinations of vaccination and re-stimulation treatment are shown on the x axis. For ‘vaccine’, Neg is the negative control group, MT is rAdMT vector control group and Env and NS3 are for the groups vaccinated with rAdE and rAdNS3 respectively. The ‘Re-Stim’ indicates the re-stimulation treatment applied to the cells ex-vivo, and NS, E3 and WN denote the negative control (no treatment), E/3 and WNV antigen re-stimulation treatments respectively. The asterisks, just above the x axis, denotes any significant difference between that vaccine/re-stimulation combination and the corresponding vaccine/no re-stimulation treatment. *** indicates a P value of <0.001, ** a P value of <0.01, * a P value of <0.05 and ‘o’ a P value of<0.1. For all plots, the 2 points on each vertical line indicate T cell numbers from the 2 individual birds, used at that time point, from each of the 4 vaccination groups; where there appears to be only 1 point on a vertical line the values for each bird are very similar. On plots for day 43 there are 2 points for each bird to reflect the 2 re-stimulation treatments used and on plots for day 93 there are 3 points for each bird to reflect the 3 re-stimulation treatments used. (DOC) [file pone.0067137.s001.doc]

**Supporting Information, Figure S1: Graphical Output of Raw Data Obtained From R Script.**


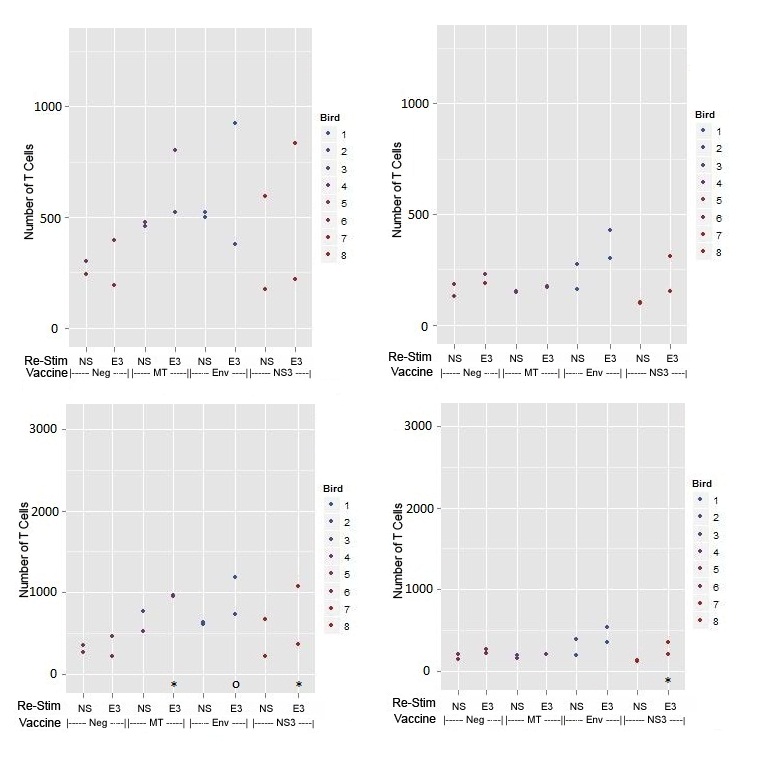


**Day 43 PB**

**CD4+IFNg+**

**Day 43 PB**

**All CD4+**

**Day 43 PB**

**CD8+IFNg+**

**Day 43 PB**

**All CD8+**

**Day 93 PB**

**CD4+IFNg+**

**Day 93 PB**

**CD8+IFNg+**


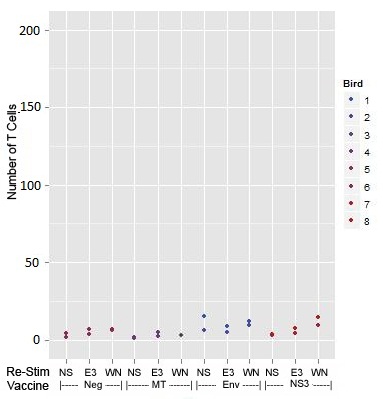

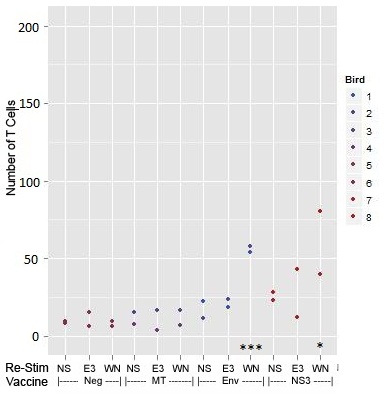

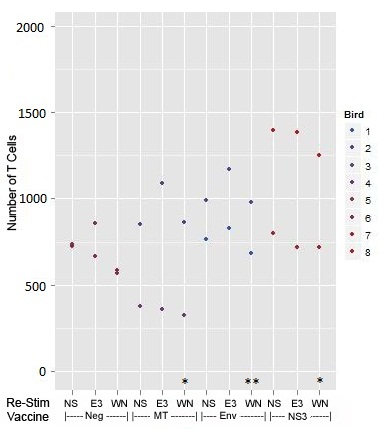

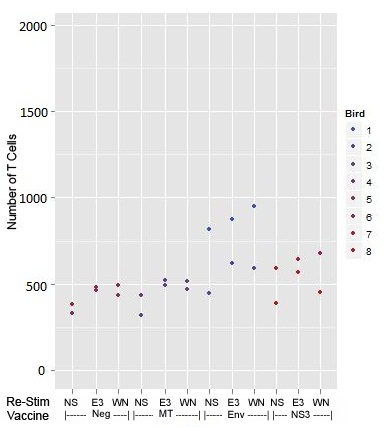


**Day 93 PB**

**CD8+IFNg+**

**Day 93 PB**

**All CD4+**

**Day 93 PB**

**All CD8+**
