# Supplementary figures and images for: Towards the Conservation of Endangered Avian Species: A Recombinant West Nile Virus Vaccine Results in Increased Humoral and Cellular Immune Responses in Japanese Quail (Coturnix japonica)
Source: PLoS One. 2013 Jun 25;8(6):e67137. doi: 10.1371/journal.pone.0067137 (PMC3692427; doi:10.1371/journal.pone.0067137)

**Supporting Information, Protocol S1: R Script Used To Assess T Cell Numbers.**


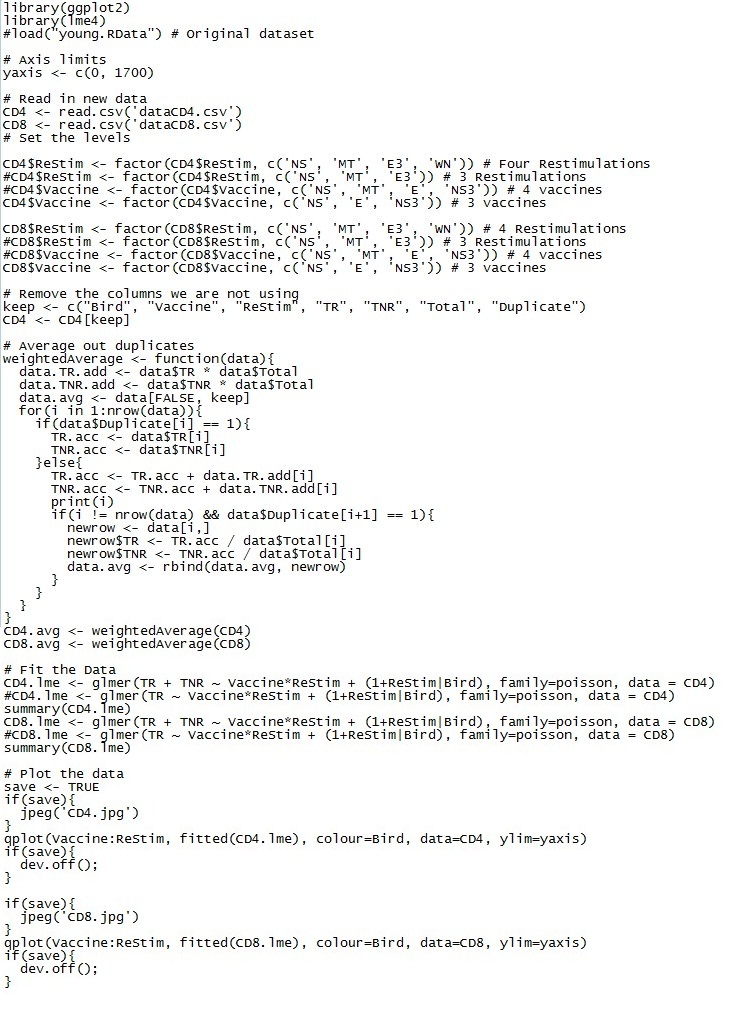

Supplement: Protocol S1 — R Script Used To Assess T Cell Numbers. The R script specially written to assess the numbers of T cells from all samples is shown below. For all cell types assessed, it compared cell numbers in re-stimulated samples to those in the no re-stimulation sample for individual birds in each experiment. The script used a generalised linear mixed model, using Poisson regression and taking into account both fixed and random effects on the data. Output from the script included calculations of P values for each comparison and plots of T cell numbers. Within the script certain lines were edited as appropriate: axis limits for the plots were set to suitable values to allow all data points, and the differences between them, to be seen clearly and for easier comparisons between cells types; whether 3 or 4 re-stimulation treatments had been used; whether there were 3 or 4 vaccine groups being analysed and whether the plots would show IFN-γ positive T cells only (denoted TR for T cell responding) or all T cells (TR cells plus IFN-γ negative cells, denoted TNR for T cell not responding). (DOC) [file pone.0067137.s002.doc]
